# The Association of High-Molecular-Weight Hyaluronic Acid (HMWHA), Alpha Lipoic Acid (ALA), Magnesium, Vitamin B6, and Vitamin D Improves Subchorionic Hematoma Resorption in Women with Threatened Miscarriage: A Pilot Clinical Study

**DOI:** 10.3390/jcm13030706

**Published:** 2024-01-25

**Authors:** Giuseppina Porcaro, Antonio Simone Laganà, Isabella Neri, Cesare Aragona

**Affiliations:** 1 Women’s Health Centre, USL Umbria 2, 05100 Terni, Italy; 2 Unit of Obstetrics and Gynecology, “Paolo Giaccone” Hospital, Department of Health Promotion, Mother and Child Care, Internal Medicine, and Medical Specialties (PROMISE), University of Palermo, 90127 Palermo, Italy; 3 Department of Medical and Surgical Sciences for Children & Adults, University of Modena and Reggio Emilia, 41124 Modena, Italy; 4 SBG Lab (Systems Biology Group), 00161 Rome, Italy

**Keywords:** threatened miscarriage, HMWHA, subchorionic hematoma, resorption, vaginal bleeding

## Abstract

Background—We evaluated whether the oral intake of high-molecular-weight hyaluronic acid (HMWHA) in association with alpha lipoic acid (ALA), magnesium, vitamin B6, and vitamin D can improve the resorption of subchorionic hematoma in cases of threatened miscarriage. Methods—In this study, we enrolled 56 pregnant women with threatened miscarriage (i.e., subchorionic hematomas, pelvic pain/uterine contractions, and/or vaginal bleeding) between the 6th and the 13th week of gestation. They were treated with vaginal progesterone (200 mg/twice a day) (control group; *n* = 25) or vaginal progesterone plus oral 200 mg HMWHA, 100 mg ALA, 450 mg magnesium, 2.6 mg vitamin B6, and 50 mcg vitamin D (treatment group; *n* = 31; DAV^®^-HA, LoLi Pharma srl, Rome, Italy). An ultrasound scan was performed at the first visit (T0) and after 7 days (T1) and 14 days (T2) until hematoma resorption. Results—At the ultrasound scan, the treatment group showed faster resorption of the subchorionic hematoma compared with the control group, both at T1 (control group 140 (112–180), treated group 84 (40–112), *p* < 0.0031), and T2 (control group: 72 (48–112), treated group: 0 (0–0), *p* < 0.0001). Moreover, subjective symptoms, such as vaginal bleeding, abdominal pain, and uterine contractions, showed a faster decrease in the treatment group than in the control group. Conclusions—The association may more rapidly improve the resolution of threatened miscarriage and related symptoms compared to the standard local protocol.

## 1. Introduction

Threatened miscarriage is a widespread early and common pregnancy complication, occurring in about 20–25% of pregnancies [1], and unfortunately can lead to early pregnancy loss.

It is estimated that about one-fifth of pregnant women with threatened miscarriage have a sub-chorionic hematoma, defined as a collection of blood between the chorionic membrane and the uterine wall [2]. Small and medium-sized subchorionic hematomas typically regress, while large ones can become larger and strip at least 30–40% of the placenta away from the endometrium, resulting in compressing the gestational sac and promoting spontaneous abortion [3]. The etiopathogenesis of SCH is yet to be fully elucidated; however, immunological factors, autoimmune diseases, pre-existing medical conditions, infections, history of recurrent pregnancy loss, and uterine malformations are believed to be predisposing factors [4]. Moreover, it seems that SCH is correlated with abnormalities of the maternal immune regulation, associated with the dysfunction and dysregulation of the cytokines expression at the maternal–fetal interface, which can affect the crosstalk between the maternal decidual stromal cells and the fetal trophoblastic cells [4]. A persistence of SCH may correlate with subsequent adverse pregnancy outcomes, including not only early and late pregnancy loss but also preterm labor (PTB) and premature rupture of membranes (PROM). Therefore, faster resolution of SCH is necessary to improve pregnancy outcomes [5,6].

Currently, no specific therapy is available to treat SCH; therefore, new strategies are necessary to improve the clinical outcomes.

Nutrition may have an important role in supporting physiological pregnancy, and several nutrients, such as alpha lipoic acid (ALA), magnesium, vitamin B6, and vitamin D, are often required and recommended to support pregnancy [7,8,9], even in the case of SCH [10,11].

Hyaluronic acid (HA) is the major non-protein component of the extracellular matrix (ECM) and consists of repeating disaccharide chains of N-acetyl-glucosamine and glucuronic acid [12]. Many of HA’s effects are orchestrated by binding to one or more specific cell receptors or by forming a complex extracellular matrix incorporating other proteoglycans/proteins [13].

Despite its simple linear structure, HA has multiple functions correlated with its molecular weight, which can vary from very low molecular weight (<10 kDa), low molecular weight (100–500 kDa), to high molecular weight (>500 kDa) [14]. While low-molecular-weight hyaluronic acid has pro-inflammatory properties [15], HMWHA demonstrates anti-inflammatory properties [16]. In recent years, HA has gained more attention in different branches of medicine without contraindications. Particularly, the investigation of HMWHA and its role throughout the gestational period has been an increasing area of investigation over the past 30 years [17,18,19,20]. 

HMWHA is present in various fluids and tissues of the reproductive system of humans, such as the stromal structures of the uterus and placenta; the angiogenic regions of decidua basalis; cumulus cells; cervical mucus; and oviductal, uterine, and follicular fluid [18]. Its physiological presence in the extracellular matrix of all the main organs and tissues involved in pregnancy indicates the importance of this molecule for a successful pregnancy.

The growth, proliferation, and differentiation of endometrial cells in the first phase of pregnancy represent the beginning of decidualization. This process, governed by LH and progesterone, is the result of significant changes in endometrium cells in preparation for pregnancy [19].

In the literature, there is no direct correlation between HMWHA and subchorionic SCH resorption. Nevertheless, as aforementioned, SCH can be correlated with abnormalities of the maternal immune regulation associated with the dysfunction and dysregulation of the cytokines expression at the maternal–fetal interface, which can affect the crosstalk between the maternal decidual stromal cells (DSCs) and the fetal trophoblastic cells (EVT). A study by Yang et al. observed, for example, a higher proportion of autoantibodies in women with SCH than in those without (45.35% vs. 21.51%, *p* = 0.000) [20].

Both DSCs and EVT express HA, and previous studies observed a low expression of HMWHA in DCSs and EVT obtained from miscarriages, compared with the levels of HMWHA observed in normal pregnancies [21,22].

HMWHA is highly expressed in the early stages of pregnancy and plays a role in the formation of the DSC maternal interface, where it stimulates immunomodulation by cell differentiation (e.g., T-naïve into Treg) [23] and decidual macrophages into M2 by CD44 activation of PI3K/Akt-STAT-3/STAT-6 signaling pathways [22]. Moreover, HMWHA induces the secretion of anti-inflammatory cytokines, such as IL-10, or inhibits the expression of pro-inflammatory factors, such as TNF-α or IFN-γ [24], which are usually associated with a higher risk of threatened miscarriage [25].

Therefore, the aim of the study was to investigate if the use of oral HMWHA in association with ALA, magnesium, vitamin B6, and vitamin D (whose combination has already been tested in the case of SCH [11]) can improve the outcome of pregnancies with threatened miscarriage by promoting subchorionic hematoma resorption.

## 2. Materials and Methods

The study was conducted as a prospective, open-label, randomized controlled study. This clinical trial was a sub-pilot study of the ClinicalTrials.gov study identification number NCT04874285, approved by the Ethics Committee Alma Res (reference number n. 05/2021). This study was carried out between May 2021 and September 2022, and all procedures were in accordance with Good Clinical Practice guidelines and the Declaration of Helsinki.

**Figure 1:**
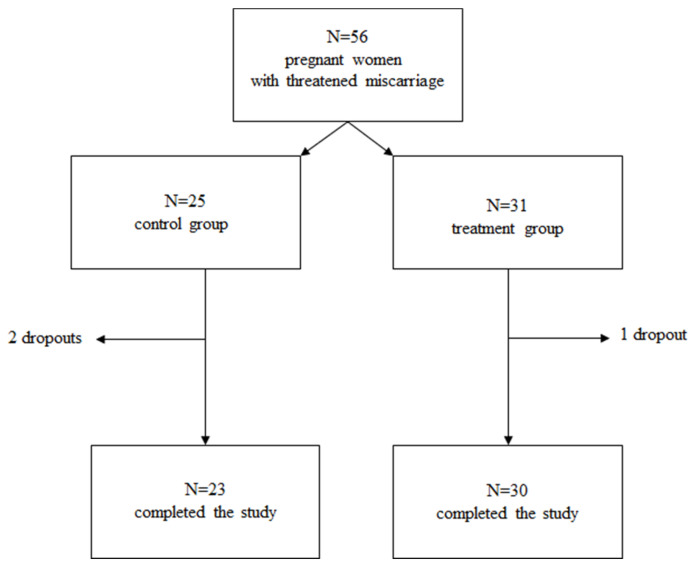
Flowchart of the study. A total of 56 pregnant women with threatened miscarriage, maternal age ≥18 and ≤50 years, and gestational age between 6th and 13th week were enrolled in this study. The control group (*n* = 25) was supplemented with vaginal suppositories of P4 (200 mg twice a day); the treatment group (*n* = 31) was supplemented with vaginal suppositories of P4 (200 mg twice a day) + HMWHA (200 mg) in combination with ALA (100 mg), magnesium (450 mg), vitamin B6 (2.6 mg), and vitamin D (50 mcg). A total of 3 patients discontinued the study (2 in the control group and 1 in the treatment group because of miscarriage). A total of 53 patients (*n* = 23 in the control group and *n* = 30 in the treatment group) completed the study and were included in the final analysis.

The enrolled subjects were randomized into two groups according to a 1:1 ratio through a generator of random and unrepeated numbers that produced different numeric sequences with initials specific to both the study arms. To avoid the lack of blindness affecting the study and representing a limit to its success, the randomization was carried out by a physician who did not participate in the study nor had contact with any patient. Regarding sample size, since no prior literature has assessed the role of HMWHA in association with ALA, magnesium, vitamin B6, and vitamin D in SCH, we devised this study as a pilot trial. Patients with the following inclusion criteria were enrolled: (i) singleton pregnancy, (ii) maternal age of ≥18 and ≤50 years, (iii) gestational age between the 6th and 13th week, and (iv) threatened miscarriage. Patients with the following exclusion criteria were excluded from the study: (i) multiple pregnancy, (ii) the presence of gestation pathologies (arterial hypertension, maternal autoimmune diseases, antiphospholipid syndrome), and (iii) treatment with anti-hypertensive and anti-coagulant drugs. A total of 56 pregnant women who were eligible according to inclusion criteria and who agreed to participate in the study were enrolled. All patients provided written informed consent. Patients in the control group (*n* = 25) included pregnant women supplemented with vaginal suppositories of P4 (200 mg twice a day) as standard local protocol; patients in the treatment group (*n* = 31) included pregnant women supplemented with the same dosage of P4 (200 mg twice a day) plus oral HMWHA (200 mg) in combination with ALA (100 mg), magnesium (450 mg), vitamin B6 (2.6 mg), and vitamin D (50 mcg) (DAV^®^-HA, Lo.Li pharma s.r.l, Rome, Italy). At least three ultrasounds were performed for each woman—at the time of diagnosis of SCH (T0), after 7 days from the first ultrasound scan (T1), and after 14 days from the first ultrasound scan (T2)—and scans continued until the complete resolution of SCH. A total of 3 patients discontinued the study (2 in the control group and 1 in the treatment group because of miscarriage). A total of 53 patients (*n* = 23 in the control group and *n*= 30 in the treatment group) completed the study and were included in the final analysis (Figure 1).

### 2.1. Sonographic Visualization of a Subchorionic Hematoma

All images of the SCH were obtained using a GE Voluson S8 ultrasound system with transvaginal probe GE (frequency range of 4.0–10.0 MHz) (GE HealthCare, Milan, Italy). SCH was defined as a fluid collection visualized on ultrasound between the gestational sac and the uterine wall. Hematoma resorption during the treatment was calculated by measuring the size of the hematoma at T0 and T1, with the average values compared. During the ultrasound appointment, patients were routinely asked about the presence of vaginal bleeding or cramping symptoms. The ultrasounds were performed by a physician who did not participate in the study and did not usually have contact with patients, thus avoiding biases.

### 2.2. Primary and Secondary Outcomes

The primary outcome of the study was the reduction/disappearance of subchorionic hematoma. The secondary outcome was the reduction of maternal subjective symptoms such as pelvic pain, vaginal bleeding, and uterine contractions.

## 3. Statistical Analysis

Data analysis was performed using SAS^®^ (Version 9.4; SAS Institute Inc., Cary, NC, USA). Descriptive analyses of quantitative data are presented in means of mean, standard deviation, standard error, median, 25th and 75th percentiles, minimum, and maximum. Qualitative variables are presented in terms of frequency distributions. Pearson’s Chi-square or Fisher’s Exact Test was applied to analyze the different % composition of qualitative variables related to symptoms between the two groups; McNemar’s Test was used to evaluate the different % composition over time of qualitative variables related to symptoms; because of the non-normality distribution of quantitative variables, the Mann–Whitney test was used to compare the variation of the extension of SCH between groups; Wilcoxon signed rank sum test was used to analyze variation of hematoma resorption over time compared to T0. The level of statistical significance was set below *p* = 0.05. Data for hematoma resorption are presented in the text as median value [25th percentile–75th percentile].

## 4. Results

A total of 56 pregnant women with a gestational age between the 6th and 13th week and threatened miscarriage were enrolled at the beginning of the study. There was no statistically significant difference between the treatment group and the control group in terms of demographic and baseline characteristics, such as age, weight, gestational age, previous cesarean section, and previous miscarriages (Table 1).

At T0, the extension of the SCH was comparable between cases and controls.

Between T1 and T2, there were three total dropouts because of miscarriage: two cases in the control group and one case in the treatment group. The incidence of miscarriage was higher in the control group compared to the treatment group; however, this was not statistically significant. (8% vs. 3.2%, respectively).

**Figure 2:**
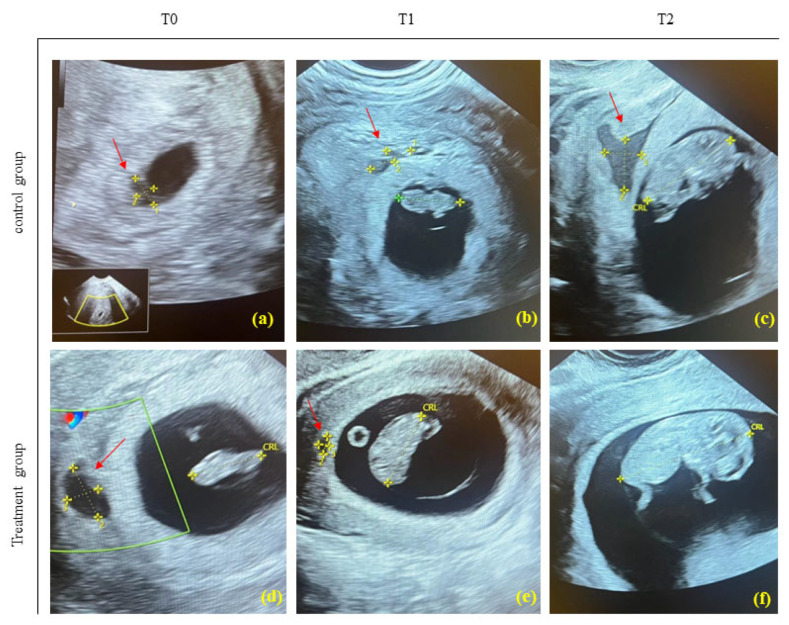
Representative ultrasounds of SCH (red arrow) of patients following or treatment with P4 (control group) (**a**–**c**) and HMWHA in association with ALA, magnesium, vitamin B6, and vitamin D (treatment group) (**d**–**f**). Ultrasound at T0, baseline; at T1 (1 week of treatment); at T2 (2 weeks of treatment). In the treatment group with HMWHA in association with ALA, magnesium, vitamin B6, and vitamin D, subchorionic hematoma (**f**) was no longer detectable at T2, unlike the control group (**c**). CRL = Crown-Rump Length.

**Figure 3:**
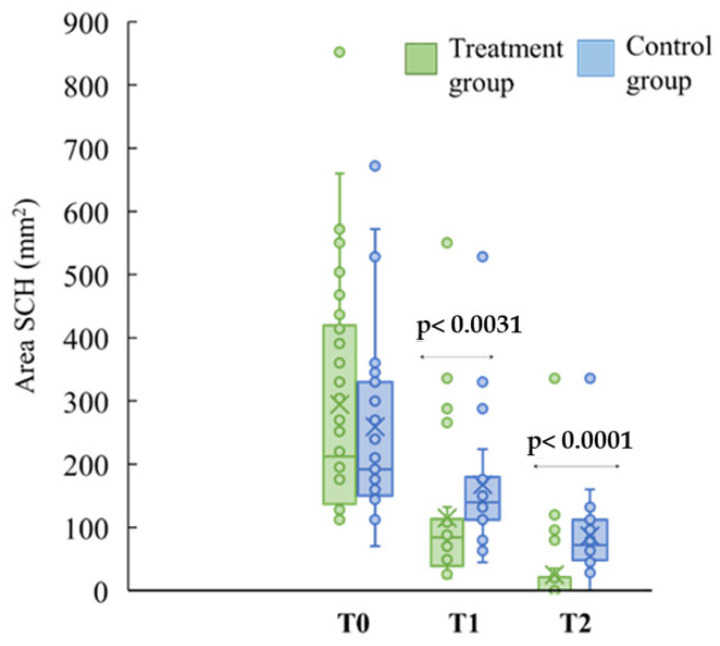
Area (mm^2^) of hematoma resorption (SCH). The progress of subchorionic hematoma resorption was detected via ultrasound in the control group (*n* = 23; in light blue) and the treatment group (*n* = 30; in green) at different time points (T0, T1, T2). Mann–Whitney test was used to analyze variation in hematoma resorption between the group at T1 (*p <* 0.0031) and at T2 (*p <* 0.0001).

The improvement of SCH significantly occurred in both groups over the time compared to T0, as shown in Figure 2, but the time required for healing was different: the treatment group (HMWHA in association with ALA, magnesium, vitamin B6, and vitamin D) exhibited a significant and faster reduction of the subchorionic hematoma area (mm^2^) compared with the control group both at T1 (control group 140 (112–180), treated group 84 (40–112), *p <* 0.0031) and T2 (control group: 72 (48–112), treated group: 0 (0–0), *p* < 0.0001) (Figure 3).

Concerning the symptoms such as vaginal bleeding, abdominal pain, and uterine contractions, the changes in these parameters from the baseline (T0) to the following medical controls (T1, T2) are shown in Table 2.

## 5. Discussion

Our study highlighted that in case of threatened miscarriage, the oral supplementation of HMWHA in association with ALA, magnesium, vitamin B6, and vitamin D induces a more rapid SCH absorption compared to only vaginal suppositories of P4 at both T1 (*p <* 0.0031) and T2 (*p <* 0.0001). Moreover, related symptoms, such as vaginal bleeding (43% of the control group vs. 3% of the treatment group, *** *p* < 0.001 at T1), abdominal pain (61% of the control group vs. 30% of the treatment group, * *p* < 0.05 at T1; 30% of the control group vs. 3% of the treatment group, ** *p* < 0.01 at T2), and uterine contractions (91% of the control group vs. 40% of the treatment group, *** *p* < 0.001 at T1) decreased faster and disappeared in the treatment group compared with the control group, thus improving quality of life of pregnant women.

Recently, vaginal and/or oral administration of ALA in association with vitamin D, magnesium, and vitamin B6 was used to improve the outcome of pregnancy in women with SCH [10,11]. It was observed that in the treatment group, the speed of resorption of subchorionic hematoma was significantly superior compared to controls. The treatment decreased all symptoms more rapidly and, in some cases, completely removed all symptoms compared to those observed in the control group.

ALA is a natural antioxidant, anti-inflammatory, and immunomodulatory molecule that decreases the secretion of inflammatory cytokines (TNF-α, IL-1β, and IL-17) and stimulates the release of the anti-inflammatory cytokine IL-10 [26,27].

Vitamin D induces a wide range of beneficial effects on pregnancy outcomes and preservation of the uterine quiescence because of its immunological actions [28]. Magnesium is one of the ten essential metals in humans, and its supplementation during pregnancy correlates with a reduced risk of fetal growth restriction and preeclampsia [29]. Vitamin B6 supplementation during pregnancy and lactation is common practice, and it has been demonstrated that its supplementation reduces nausea and vomiting in pregnant women [30].

Despite its role in female reproductive biology and the scientific evidence regarding its supportive role during gestation [31], oral HMWHA has been poorly investigated as a food supplement in pregnant women. The absorption of HA remains still controversial since it is difficult to estimate its distribution throughout the entire body accurately, and some studies have different results [32,33]. However, it was speculated that while smaller molecules of HA are more easily absorbed, larger molecules of HA are absorbed mainly through the portal vein and delivered intact to the gut-associated lymphatic tissue via the M cells [34,35]. Upon its absorption, HMWHA may exercise two supportive effects in combination with ALA, magnesium, vitamin B6, and vitamin D. First, HA may exercise a molecular-weight-dependent modulation of the mucin nanostructure, which may contribute to the resorption of ALA, magnesium, vitamin B6, and vitamin D [36]; second, HA may also act as a regulatory factor of the gut microbiome [37,38], whose homeostasis has an essential influence on the entire body and whose unbalances is accompanied by a differential alteration in epithelial cell expression of TLR4 involved in the innate immunity of intestinal epithelium [39].

A recent study observed that the oral administration of HMWHA during gestation seems to be associated with improvement in pregnancy outcomes [40]. In this observational retrospective study, data from approximately 250 pregnant women, aged between 25 and 41 years old and at the 7th gestational week, were collected. Results clearly demonstrated that the oral supplementation of HMWHA in combination with ALA, magnesium, vitamin B6, and vitamin D could counteract adverse events in pregnancy, such as PTB, spontaneous contractions, miscarriages, and hospitalization, compared with the control group. Although the authors did not record long-term data on the outcome of the patients (mother and infant), there was no reported scientific evidence showing harmful effects or contraindications regarding the administration of HMWHA during gestation [40].

Therefore, the aim of our pilot study was to test if HMWHA in association with ALA, magnesium, vitamin B6, and vitamin D could be more beneficial in the case of threatened miscarriage by inducing a SCH absorption compared to a standard treatment protocol with P4.

HMWHA is essential in female reproductive biology, from folliculogenesis to birth. It forms a hydrated and viscoelastic matrix around the oocyte in the ovarian follicles [41], maintains fetal membrane integrity, and is involved in several physiological processes required for a successful pregnancy, such as implantation, immune response, uterine quiescence and cervical remodeling, thus improving pregnancy outcomes [31]. Furthermore, a deficiency in HA has been linked to a greater risk of ascending infection and PTB, and thus, HA supplementation may be protective against infection-mediated preterm birth [42].

HMWHA may also support P4 activity by increasing the expression of a specific P4 receptor known as the Progesterone Receptor Component 1 (PGRMC1) [43], a non-classical progesterone receptor that is upregulated during pregnancy and downregulated near delivery [44] or in pathological gestational conditions [45,46].

Wu et al. conducted an in vitro study in myometrial tissues and a human myometrial cell line, where it was observed that PGRMC1 was expressed in plasma and nuclear membranes in addition to the cytoplasm, suggesting an important function at the maternal–fetal interface. Furthermore, through the use of specific PGRMC1 antibodies, it was observed to modulate P4 activity in myometrial tissue [44]. Additionally, PGRMC1 expression is upregulated in pregnant women and downregulated during term and preterm labor, suggesting a vital role during pregnancy. Moreover, the importance of PGRMC1 in mediating P4 signaling is evidenced by the fact that women who suffered from recurrent miscarriages (RM) and PTB have decreased PGRMC1 expression [47,48].

The expression of PGRMC1 allows to preserve uterine quiescence until labor. It is not by chance, for example, that in the treatment group, pelvic pain, and spontaneous contractions were significantly reduced compared with the control group (Table 2).

It should be noted that the limited number of evaluated patients can represent a limitation of the study. The population represented in the study were Italian Caucasian women between the ages of 26 and 38. It is, therefore, possible that the results described herein may not apply to other population groups with threatened miscarriage. Therefore, other blinded studies with larger sample sizes will be useful to corroborate the reported evidence and strengthen the use of these natural molecules in the management of patients with threatened miscarriage in the presence of SCH.

## 6. Conclusions

This pilot study highlighted that HMWHA in association with ALA, magnesium, vitamin B6, and vitamin D may play a pivotal role in ameliorating medical conditions of pregnant women with threatened miscarriage. The presence and persistence of SCH can impair the outcome of pregnancy, thus inducing early pregnancy loss. Although the etiopathogenesis of SCH is not fully elucidated, an imbalance and alteration of immunity and anti-/pro-inflammatory cytokine equilibrium at the maternal–fetal interface seems to be one of the factors that can promote SCH. In this regard, the association of HMWHA, ALA, magnesium, vitamin B6, and vitamin D may represent an innovative approach to support physiological pregnancy in women with threatened miscarriage by inducing a faster SCH resorption. Future and larger studies are necessary to deepen our preliminary results.

## Figures and Tables

**Table 1:** Baseline demographics and clinical characteristics. Data are expressed as number (percentage) or mean ± standard deviation. No significant difference was observed between the two groups (*p* > 0.05).

	Control Group (*n* = 25)	Treatment Group (*n* = 31)	*p* Value
Age (years)	32.0 ± 2.5	32.6 ± 3.8	0.46
Weight (kg)	61.2 ± 6.8	61.2 ± 7.3	0.99
Gestational age (weeks)	8.3 ± 1.0	8.6 ± 0.9	0.41
Parity	11 (44%)	16 (52%)	0.60
Previous cesarean section	3 (12%)	4 (13%)	1.00
Previous miscarriage	8 (32%)	9 (29%)	1.00

**Table 2:** Effects of treatments on symptoms of threatened miscarriage. Data are given as sample size (number and percentage) of each group at different time points: T0, baseline medical examination; T1, 1 week after baseline medical examination; T2, 2 weeks after baseline medical examination.

Symptoms	T0		T1		T2	
	Ctrl Group *n* (%)	Treat. Group *n* (%)	Ctrl Group *n* (%)	Treat. Group *n* (%)	Ctrl Group *n* (%)	Treat. Group *n* (%)
Vag. bleed.	15 (65%)	14 (47%)	10 (43%) #	1(3%) *** ###	2 (8.7%) ###	0
Abd. pain	23 (100%)	30 (100%)	14 (61%) ##	9 (30%) * ###	7 (30%) ###	1 (3%) ** ###
Uter. Contr.	23 (100%)	30 (100%)	21 (91%)	12 (40%) *** ###	12 (52%) ###	0

Abbreviation: ctrl = control; vag. bleed. = vaginal bleeding; abd. = abdominal, uter. contr. = uterine contractions. * *p* < 0.05, ** *p* < 0.01, *** *p* < 0.001 vs. ctrl (Fisher’s Exact Test). # *p* < 0.05, ## *p* < 0.01 ### *p* < 0.001 vs. T0 (McNemar’s Test).

## Data Availability

The data presented in this study are available on request from the corresponding author.
